# The Challenge of Managing Neuropathic Pain in Children and Adolescents with Cancer

**DOI:** 10.3390/cancers17030460

**Published:** 2025-01-29

**Authors:** Flaminia Coluzzi, Giulia Di Stefano, Maria Sole Scerpa, Monica Rocco, Giovanni Di Nardo, Alice Innocenti, Alessandro Vittori, Alessandro Ferretti, Andrea Truini

**Affiliations:** 1 Department of Surgical and Medical Sciences and Translational Medicine, Sapienza University of Rome, 00189 Rome, Italy; monica.rocco@uniroma1.it; 2 Unit Anesthesia, Intensive Care and Pain Therapy, Sant’Andrea University Hospital, 00189 Rome, Italy; 3 Department of Human Neuroscience, Sapienza University, 00189 Rome, Italy; giulia.distefano@uniroma1.it (G.D.S.); andrea.truini@uniroma1.it (A.T.); 4 Department of Neurosciences, Mental Health and Sensory Organs (NESMOS), Sapienza University of Rome, 00189 Rome, Italy; giovanni.dinardo@uniroma1.it (G.D.N.); alessandro.ferretti@uniroma1.it (A.F.); 5 Pediatric Unit, Sant’Andrea University Hospital, 00189 Rome, Italy; 6 Unit of Child Neurology and Psychiatry, Department of Human Neuroscience, Sapienza University of Rome, 00189 Rome, Italy; alice.innocenti@uniroma1.it; 7 Department of Anesthesia and Critical Care, ARCO ROMA, Paediatric Hospital Bambino Gesù IRCCS, 00189 Rome, Italy; alexvittori@libero.it

**Keywords:** neuropathic pain, cancer, children, chemotherapy-induced peripheral neuropathy, opioids, gabapentinoids, antidepressants, pediatric pain

## Abstract

Neuropathic pain (NP) is a common complication associated with some types of childhood cancer, mainly due to nerve compression, chronic post-surgical pain, chemotherapy, and radiotherapy. NP is usually less responsive to traditional analgesics, and there is generally a lack of evidence on its management in cancer patients, leading to recommendations often based on clinical trials conducted on other forms of non-malignant NP. In pediatric oncology, managing NP is still very challenging for physicians. Different factors contribute to increasing the risk of undertreatment: (a) children may be unable to describe the quality of pain; therefore, the risk for NP to be underestimated or remain unrecognized; (b) specific tools to diagnose NP have not been validated in children; (c) there is a lack of randomized clinical trials involving children, with most evidence being based on case series and case reports; (d) most drugs used for adult patients are not approved for childhood cancers, and drug regulation varies among different countries; (e) recommendations for pediatric pain treatment are still not available. In this paper, a multidisciplinary team will review the current literature regarding children with cancer-related NP to define the best possible diagnostic strategies (e.g., clinical and instrumental tests) and propose a therapeutic care pathway, including both non-pharmacological and pharmacological approaches, which could help pediatricians, oncologists, neurologists, and pain therapists in designing the most effective multidisciplinary approach.

## Simple Summary

The management of neuropathic pain in cancer patients remains a challenge, particularly in children and adolescents. The subjectivity of pain, the limited approved drugs, the scarcity of clinical trials, and the lack of guidelines are the most relevant difficulties in assessing, evaluating, and managing neuropathic pain in children.

## 1. Introduction

According to the International Association for the Study of Pain, neuropathic pain (NP) is a chronic pain condition caused by “a lesion or disease of the somatosensory nervous system”. Therefore, an NP should be considered a clinical description of a painful condition rather than a diagnosis. A suspected diagnosis needs to be confirmed by demonstrating an actual lesion of the somatosensory nervous system, either through imaging, neurophysiology, lab tests, or biopsies, or by the evidence of an underlying disease, which is likely to be the cause of the lesion [1].

Pediatric chronic pain has an overall prevalence ranging from 5 to 6% [2]; on the other hand, NP is generally less frequent in children than in adults, although the exact prevalence is unknown [3]. Reliable data on pediatric NP are scarce, unlike adults, where painful conditions associated with NP range from trigeminal or post-herpetic neuralgia, radiculopathies, and stroke to diabetic neuropathies; pathological states causing NP are much less common in children and adolescents, and their clinical manifestations may be less plain [4]. That may be the case for traumatic lesions involving a structure of the somatosensory nervous system, as observed in complex regional pain syndrome or after spinal cord injury, which is recognized as the most common cause of NP in children and adolescents [5]. Alternatively, nerve injury may be caused by cancer as a result of the effects of the disease itself (i.e., infiltration or compression of a nerve or a plexus) or due to treatments (i.e., chemotherapy, surgery, and radiotherapy) [6]. In a small proportion of pediatric patients, NP may be caused by genetic diseases affecting sensory fibers (C and A-delta), through a metabolic dysfunction, as in Fabry’s disease, which is due to a lack of alpha-galactosidase A enzyme [7], or specific channelopathies, such as primary erythromelalgia, caused by the mutation of the encoding gene for the voltage-gated sodium channel NaV1.7 [8]. Finally, painful neuropathies in children may be related to autoimmune and degenerative diseases, such as Guillain–Barré syndrome [9] and Charcot–Marie–Tooth disease [10].

Through the involvement of a multidisciplinary panel of experts in the discussion of the currently available literature, this review aims to provide an instrument for improving the recognition and management of NP in children and adolescents with cancer. The panel included pain therapists (FC, MSS, MR, and AV), neurologists (GDS and AT), pediatricians (GDN and AF), and a pediatric neuropsychiatrist (AI).

## 2. Causes of NP in Children and Adolescents with Cancer

According to the American Cancer Society, childhood cancer is rare, but it is the second most common cause of death in children aged one to fourteen. In 2024, statistics state that one in 257 children and adolescents younger than 20 years of age will be diagnosed with cancer. Leukemia is the most common type of cancer in children aged 1 to 14, with a percentage of 28%, while central nervous system neoplasms are the most common among adolescents (aged 15 to 19), with a percentage of 22% of overall malignant diseases. The cancer mortality rate has dropped by 70% in the last fifty years, falling from 6.3% per 100,000 children in 1970 to 1.9% in 2021, probably thanks to the significant reduction in mortality from leukemia. Similarly, a 63% reduction in mortality by cancer was recorded among adolescents [11]. The increase in survival rate led to greater attention towards a growing population of childhood cancer survivors (CCSs). As of today, 85% of children diagnosed with cancer are alive after 5 years, and 80% are alive after 10 years. In 2020, there were an estimated 496,000 CCSs in the United States alone [12]. With such an increase in childhood cancer survival, addressing the side effects of cancer treatments has become a priority. Peripheral painful neuropathies have been widely described as a common complication in malignancies, either due to cancer itself or as an iatrogenic consequence of treatments. Therefore, neuropathies are a significant concern in the setting of pediatric oncology since they may limit the use of chemotherapeutics and strongly affect the quality of life (QoL) for both children with active cancer and CCSs [13], as well as in an end-of-life setting [14].

### 2.1. Cancer-Related NP in Children and Adolescents

Solid tumors, such as neuroblastoma, may cause NP in children and adolescents through infiltration and compression of peripheral nerves. Neuroblastoma arises in the sympathetic nervous system, and it is the most common extracranial solid tumor in childhood. Its incidence is higher in children (6% of all cancer diagnoses) compared to adolescents (<1%) [11]. Other common malignancies in younger patients include sarcoma [15] and osteosarcoma [16], as well as other tumors of the central nervous system [17], with all of them being possibly accompanied by neuropathic manifestations after compression and/or infiltration of nervous structures.

### 2.2. Chemotherapy-Induced NP in Children and Adolescents

Chemotherapy-induced peripheral neuropathy (CIPN) reaches a prevalence of 90% among children with cancer and CCSs, and it is one of the most common reasons for referral to pain medicine services. Pain in the upper and lower extremities is one of the sensory symptoms observed in CIPN, together with numbness, tingling, weakness, and impaired balance. The most relevant risk factors for CIPN are the type of chemotherapeutic agent and the cumulative dose; vinca alkaloids and platinum are primarily involved in CIPN [18]. For instance, in young patients with leukemia, vincristine-induced peripheral neuropathy (VIPN) is the most common cause of NP [19]. Vincristine binds to microtubules of the cell cytoskeleton, disrupts axonal transport, and induces axon degeneration; moreover, it alters the mitochondrial electron transport chain [20]. VIPN is usually distal and symmetrical, with a typical “socks and gloves” distribution. Paresthesia, numbness, and tingling may progress proximally and progressively involve larger nerve fibers. Autonomic symptoms, such as constipation and dizziness, may be observed in a few cases.

Classification of CIPNs is not easy, given their subjective nature, particularly in children. Most clinical trials reported the incidence of CIPNs according to the World Health Organization’s (WHO) Common Toxicity Criteria for Peripheral Neuropathy, which categorizes CIPN into four grades, as follows: (1) Grade 1: paresthesia and decreased tendon reflexes; (2) Grade 2: severe paresthesia and mild weakness; (3) Grade 3: intolerable paresthesia and marked motor loss; (4) Grade 4: paralysis [21]. Data on CIPN prevalence are available for most hematological and solid tumors, while it may be difficult to specifically extrapolate the prevalence of NP in the context of CIPN. According to the WHO grading system, VIPN of any grade accounts for 78 to 100% of children with leukemia, while Grade 3 or 4 have been described in 10–50% of them [18]. Since there are no validated strategies to reduce the incidence of VIPN, physicians often tend to reduce the dose of vincristine to mitigate moderate-to-severe VIPN [22]. Similarly, nelarabine, a nucleoside analog used for the treatment of patients with T-cell acute lymphoblastic leukemia (T-ALL)/lymphoma, has been associated with persistent peripheral and central neurotoxicity [23].

Oxaliplatin and carboplatin have been studied to treat relapsed solid tumors, low-grade glioma, and retinoblastoma in children: they were correlated with an incidence of Grade 1/2 CIPN ranging from 37% to 50% with oxaliplatin and about 4% with carboplatin [24]. Cisplatin is commonly used as an adjuvant drug in the treatment of many childhood solid malignancies, such as osteosarcoma and hepatocellular carcinoma, and it is well known to induce CIPN, ototoxicity, nephrotoxicity, or hepatotoxicity [25]. Cisplatin increases the production of intracellular reactive oxygen species (ROS) and alters mitochondrial function. Overall, platinum-based antineoplastic drugs prevent DNA replication and transcription, leading to cell apoptosis [18]. Other chemotherapeutic agents involved in CIPN development among children include bortezomib, which reversibly inhibits the 26S proteasome and has a therapeutic application for children in T-ALL, in association with nelarabine [26]. The mechanism is still not completely understood, but bortezomib is well known to impair mitochondrial function and increase ROS production, which sensitizes transient receptor potential cation channel subfamily A member 1 (TRPA1), resulting in sensory-predominant peripheral neuropathy. Despite the high incidence of Grade 1/2 CIPN, bortezomib has been proposed as a substitute for vincristine in ALL treatment as a potential strategy to mitigate severe VIPN [27].

CINP may also affect children diagnosed with neuroblastoma undergoing infusions of dinutuximab, an antibody targeting the disialoganglioside GD2, which is highly expressed in neuroblastoma cells. Dinutuximab-induced neuropathic pain is supposed to be mediated by the interaction of the antibody with the GD2-expressing nerve fibers, particularly C-fibers. Pain occurs in 88% of patients during the infusion of immunotherapy, only to stop shortly after the termination of the injection, and is usually located in extremities, as well as the abdomen, chest, and back [28]. Ganglioside GD2 antibodies induce mechanical allodynia but not hyperalgesia [29]. Brentuximab vedotin is an agent used for Hodgkin’s lymphoma (HL) CD30+, and it acts by secreting conjugated microtubule-disrupting agent monomethyl auristatin E (MMAE) against CD30 cancer cells. More than 70% of patients may develop brentuximab-induced neuropathy, which is believed to be reversible after discontinuation of treatment [30].

### 2.3. Radiotherapy-Induced NP in Children and Adolescents

Radiotherapy (RT) plays a key role in treating many childhood cancers. However, RT may slow physiological growth, induce neurocognitive impairment, increase the risk of developing secondary malignancies, and trigger long-term side effects even in a cancer-free status, including fibrosis, dermatitis, enteritis, lymphedema, and chronic and neuropathic pain, with distal paresthesia and weakness being the most usual manifestations [31]. Childhood cancer survivors may live for decades after radiation therapy, and the latency of toxicity may be very long. Moreover, the concomitant use of chemotherapy may modify or increase the level of toxicity. For these reasons, pediatricians should be aware that monitoring the long-term outcome of radiation therapy in children is crucial for early detection of possible complications, such as chronic pain syndromes [32]. Radiotherapy is a standard line of treatment, especially in children and adolescents with brain and spinal malignancies. Therefore, the most frequent forms of radiotherapy-induced peripheral neuropathies (RIPNs) involve cranial or peripheral nerves. In this case, neuropathic pain is the result of direct nerve injury through demyelination or axonal damage or indirect nerve compression by tissue fibrosis as a response to radiation-induced inflammation. Radiation-induced side effects, including neuropathic pain, are volume- and dose-dependent. Nonetheless, the most recent irradiation techniques have significantly reduced its incidence [33,34].

### 2.4. Chronic Post-Surgical Pain in Children and Adolescents

Surgically induced neuropathic pain is related to contusion, transaction, stretching, and inflammation of nervous structures. Post-surgical neuropathic pain is highly prevalent in young patients with osteosarcoma, both after limb-sparing surgery and post-amputation, the latter eliciting phantom limb pain (PLP) [35]. PLP has been described in children and adolescents, both after non-cancer (e.g., traumatic) and cancer-related amputations. Despite it being a rare condition, PLP is associated with persistent refractory pain and disability: its prevalence is lower in children than in adult amputees, with even lower risk for younger patients and congenital amputees. However, the risk of developing PLP is significantly higher for cancer children compared to non-cancer children, with prevalence ranging from 48 to 90% [36,37]. Chemotherapy, significantly when vincristine- and cisplatin-based, and pain before amputation are strongly correlated with increased incidence and worsening of PLP [38]. More recently, data have showed that the incidence and duration of neuropathic pain are similar after amputation and limb-sparing surgery, with no significant difference in dosage and duration of pharmacological therapies. Still, prevention of neuropathic pain is pivotal, and perioperative administration of gabapentinoids may be beneficial [35].

## 3. Diagnostic Approach to NP in Children and Adolescents

**Figure 1:**
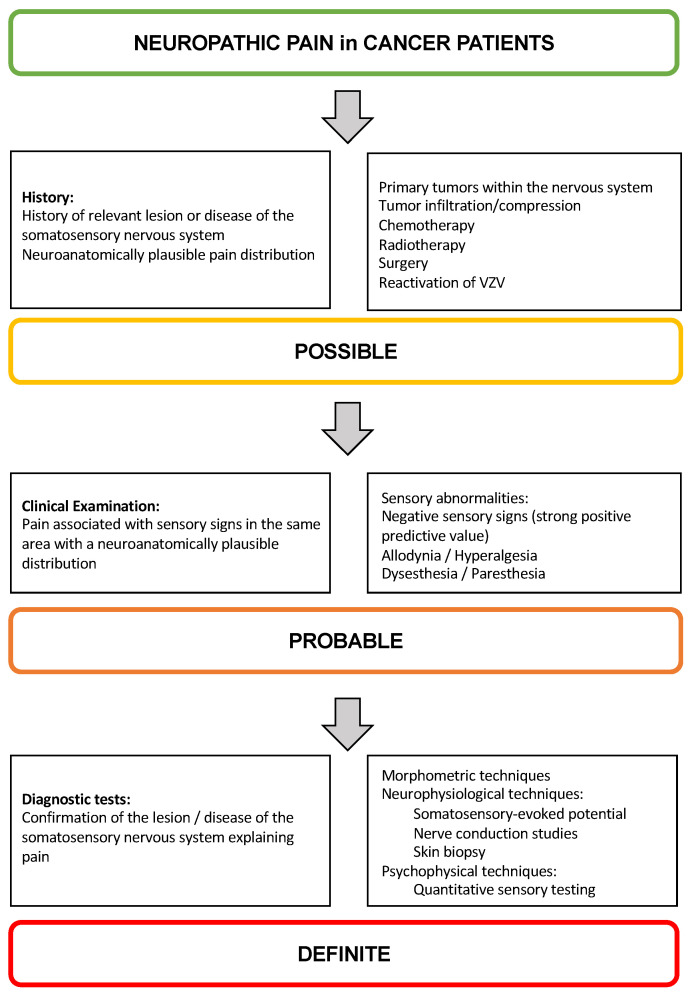
Flow chart of the grading system for neuropathic pain in cancer patients.

The diagnostic approach to neuropathic pain in children, albeit crucial to selecting the most suitable analgesic therapy, is particularly challenging, given the difficulty for children to describe the quality of pain and the lack of validated neuropathic pain tools. Therefore, because evidence in children is lacking, such diagnostic approaches are based on evidence derived from data in adults [39]. For instance, cancer may lead to a series of sequelae, such as sleep disturbances, anxiety, and depression. Whether these were secondary manifestations of cancer or, instead, an expression of uncontrolled pain should be extensively investigated [40,41]. Once pain is identified, it should be measured through appropriate scales and instruments to avoid underestimation [42,43]. A grading system guides the diagnostic process: three levels of certainty are proposed, with patient history identifying “possible” neuropathic pain, physical examination suggesting “probable” neuropathic pain, and diagnostic testing confirming the latter as “definite” [44] (Figure 1).

Possible neuropathic pain may be suspected when clinical history suggests a potential lesion or disease of the somatosensory system. In addition, pain distribution should be neuroanatomically plausible. Regarding clinical history, neuropathic pain may present in children with primary tumors within the nervous system, as well as tumor invasion or compression of neural structures from the spinal cord itself to spinal nerve roots, nerve plexuses, and peripheral nerves. As previously reported, neuropathic pain may also occur after surgery, chemotherapy, and radiation [45]. Another possible cause is represented by the reactivation of dormant varicella zoster virus infection, which should be ruled out in cancer children since immunodeficiency from cancer treatments is not uncommon.

As mentioned, validated tools for pediatric pain measurement are lacking, given the meager ability of young children to use visual analog (VAS) or numerical scales (NRS), together with the difficulty in assessing the qualitative (burning, shooting, radiating, electric shock, stabbing, pricking, tingling, pins and needles, and pinching) and temporal characteristics of pain.

The Adolescent Pediatric Pain Tool (APPT) is a self-reported, multidimensional assessment of pain intensity, location, and quality. Nonetheless, it comprises a limited amount of descriptors associated with neuropathic pain; hence, it may be useful as an initial tool for neuropathic pain assessment, but further investigation is mandatory [46]. The APPT is validated for children and adolescents aged 8 to 17. Validated scales for neuropathic pain evaluation for children under the age of five are not available. Overall evaluation of pediatric pain includes several questionnaires and instruments, namely Likert-type VAS, COMFORT scale, CRIES (Child Revised Impact of Events Scale), FLACC (Face, Legs, Activity, Cry, Consolability), and NIPS (Neonatal Infant Pain Scale) [47]. Still, their suitability for neuropathic pain has never been investigated. The very first screening tool to be validated for the diagnosis of peripheral neuropathy related to chemotherapy in children was the Pediatric-Modified Total Neuropathy Scale (ped-mTNS), validated for children aged 5 to 18 years, and based on questions related to motor, sensory, and autonomic symptoms, as well as pin sensation, light touch sensation, vibration exam, strength, and deep tendon reflexes [48,49]. The Total Neuropathy Score Pediatric Vincristine (TNS-PV) was a variation of ped-mTNS with the addition of hoarseness and constipation to the evaluation and intended for children experiencing VIPN, validated for patients ≥ 6 years of age [50]. Other specific tools, such as the Oucher Pain Scale and the Wong–Baker Faces Scale, draw a spectrum of facial expressions useful for pain assessment, but they are not valid for children younger than 3 years of age, and they are not validated for neuropathic pain assessment [51]. The possible application of the PainDETECT questionnaire to the pediatric population was first investigated in 2023 by Hess et al.; it demonstrated the ability to screen for neuropathic pain with good correlativity to quantitative sensory testing (QST) findings [52].

The next level of certainty, i.e., probable neuropathic pain, requires supporting evidence obtained by clinical examination [44]. However, sensory abnormalities are more difficult to elicit in infants and young children. Neurological examination should confirm the presence of negative sensory signs coherent with the lesion or disease of the somatosensory nervous system. The probability of neuropathic pain is much less likely with positive sensory signs alone, remarkably if their pattern does not follow a neuroanatomical distribution.

Regarding the final level of certainty, morphometric, neurophysiological, and psychophysical techniques are available for diagnosing neuropathic pain.

**Figure 2:**
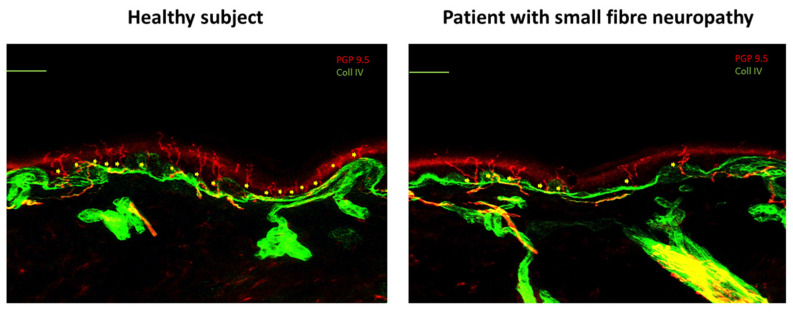
Skin biopsy. Exemplification indirect immunofluorescence skin biopsy images from a distal site show normal intraepidermal nerve fiber density in a healthy subject and reduced skin innervation in a patient with small-fiber neuropathy. Protein gene product 9.5 (PGP9.5) immunoreactive skin thermo-nociceptive fibers are marked in red. Collagen IV immunoreactive structures are marked in green. Calibration bars: 100 u.

According to a recent task force by the European Academy of Neurology (EAN), the European Pain Federation (EFIC), and the Neuropathic Pain Special Interest Group (NeuPSIG) of the IASP, the recommendation for skin biopsy is strong, while there is only a weak recommendation for quantitative sensory testing and nociceptive evoked potentials [53]. However, data on the diagnostic utility in children are still poor, with few reports in the diagnosis of small-fiber neuropathies, when standard neurophysiological tests show unremarkable findings [54]. Skin biopsy is a minimally invasive and reliable technique that quantifies nociceptive fibers in the human epidermis and dermis and diagnoses small-fiber neuropathy [55]; however, it is not routinely performed in daily practice, as the expertise for making accurate diagnosis may not be widely available. Under local anesthesia, a skin punch biopsy is collected at a distal site 10 cm above the lateral malleolus and at a proximal site 20 cm under the anterosuperior iliac spine [56]. The most commonly used marker for nerve fiber quantification is antibodies against protein gene product (PGP) 9.5, a non-specific panaxonal marker widely distributed in the peripheral nervous system [57] (Figure 2).

Among neurophysiological techniques, somatosensory-evoked potentials and nerve conduction studies are still considered the reference standard to diagnose somatosensory nervous system damage, as they are readily available and applicable; however, they do not pointedly assess the nociceptive system. The latter should be thoroughly studied through evoked potentials to nociceptive stimuli, such as contact heat or radiant stimuli, which activate nociceptors and result in contact-heat- (CHEPs) and laser-evoked potentials (LEPs), respectively [58,59]. These techniques are not commonly used in day-to-day clinical practice, but they provide evidence of minute, image-proven lesions within the nociceptive system. Hence, they are recommended when conventional neurophysiology is normal.

Quantitative sensory testing is a psychophysical measure of perception in response to mechanical, thermal, and painful stimuli of controlled intensity. This technique allows for assessing and quantifying both loss and gain of function (sensory deficits and positive signs, respectively) in the nociceptive pathways, both in peripheral nerves and the central nervous system (CNS). It represents the referential technique for identifying functional somatosensory disturbances with neuroanatomically plausible distribution in most patients with probable neuropathic pain, thus supporting the diagnosis [53]. A QST protocol was proposed by the German Research Network on Neuropathic Pain (DFNS) for neuropathic pain in adults [60], and it was later revised to be feasible and valid for children and adolescents older than 5 years of age, with specific reference values [61]. Nonetheless, QST testing requires cooperation and good cognitive functioning, thus limiting its use in children to selected cases only. Overall, recommendations for QST in diagnosing neuropathic pain are weak [53].

## 4. Managing NP in Children and Adolescents with Cancer

Management of neuropathic pain in children continues to be challenging in current clinical practice. High-quality scientific evidence is still being determined. Hence, pain treatment is still tricky [62,63]. Logistical, organizational, and ethical issues in pediatric pain research make it challenging to design randomized controlled trials (RCTs) [64]. First, clinicians must remember that several non-pharmacological techniques are available, such as physical rehabilitation, acupuncture, and mindfulness [65]; these have very few contraindications. They can be successfully integrated with pharmacological therapy, all the while possibly avoiding inauspicious interactions with patients’ anti-tumor therapy [66]. Commonly used analgesics, such as acetaminophen, dexamethasone, and opioids, are known to modulate the expression of transporters, namely P-glycoprotein, across the BBB and in several body tissues, which could impede chemotherapeutic agents from reaching and entering target organs [67].

Nonetheless, first, pharmacological approaches should always consider the possibility of mitigating pain through tumor-targeting therapies [68,69]; for instance, corticosteroids may be administered when neoplasms are compressing nervous structures, thus causing swelling and neuropathic pain. Dexamethasone may be administered at a dosage of 0.25 mg/kg IV or PO every 6 h, with standard doses ranging from 4 to 16 mg/day [70]. Painful manifestations should be targeted if this is impossible, always maintaining a mechanism-based rationale. Commonly used analgesics in children are summarized in Table 1.

Non-steroidal anti-inflammatory drugs (NSAIDs) are commonly used in acute pain in children and adolescents for their anti-inflammatory properties [71,72], e.g., in rheumatic diseases and to reduce fever. NSAIDs are not indicated for NP [73]. Moreover, they are burdened by possible side effects such as bleeding, gastric damage, and deterioration of renal function [74]. Other adverse effects in children include headache, nausea, diarrhea, constipation, dizziness, rash, and abdominal pain [72,75]. Overall, data on the safety of most NSAIDs in infants are limited. NSAIDs are not recommended for neuropathic pain, but they can be used for managing cancer pain in children by using the minimum effective dose, preferring oral formulations with a weight-based dosage, and keeping the duration of treatment as short as possible [72,76]. Among the many FDA-approved NSAIDs for children, ibuprofen is the most studied and used, and it is the only one to be approved for patients as young as 3 months by the European Medicines Agency (EMA) and 6 months by the Food and Drug Administration (FDA) [77]. The recommended dose for ibuprofen is 5 to 10 mg/kg every six to eight hours, with a maximum daily dose of 1200 mg. Currently, however, evidence on the safety and efficacy of NSAIDs in children and adolescents with chronic cancer [78] and non-cancer [79] pain is scarce.

Acetaminophen is also widely used to treat pain in children and adolescents, from the emergency setting [80] to postoperative pain [81,82], as well as serving as an antipyretic. Acetaminophen seems to have similar efficacy and safety profiles to ibuprofen in acute pain management [83,84], and the two may also be administered in fixed-dose combination to minimize relative dosage and hence the risk of adverse effects. Evidence on the effectiveness of acetaminophen in chronic pain and palliative care in children is lacking [85]; moreover, it is not included as a first-choice therapy for NP. Acetaminophen is often used as add-on therapy for its sparing effect on other analgesic drugs. The recommended dosage in children is 10 to 15 mg/kg every six hours. According to age, the maximum suggested dose is 60 mg from 6 to 12 months old and 1000 mg for children over 12 years old [86]. In the last few years, some concerns have been raised about the safety profile of acetaminophen in long-term use in children, with special regard to those at risk for liver and kidney failure [83]. Finally, despite some reports on the potential dangers of neurodevelopment impairment in susceptible babies and children exposed to acetaminophen, especially during pregnancy or in the early post-partum period [87], there is no evidence from RCTs to support or refute its use in pediatric age. Acetaminophen continues to be widely used in pregnancy [88], and it is the only recommended analgesic for babies under 3 months of age [86].

According to IASP guidelines, anticonvulsants and antidepressants are first-line treatments for neuropathic pain, regardless of the underlying disease [73]. Even in the pediatric population, gabapentinoids, namely gabapentin and pregabalin, and antidepressants, such as amitriptyline or nortriptyline, are administered as first-line therapy drugs for neuropathic pain [49] starting from the early stages through to end-of-life settings [89]. The use of gabapentinoids is accepted mainly for adults with both cancer [68] and non-cancer neuropathic pain [90]. Their use in the pediatric population is essentially based on the literature evidence derived from adults [91], granted that doses must be adapted in children compared to adult dosage [92]. However, definite data about dose rationale in children are lacking. For gabapentin, Healy et al. proposed a T.I.D. administration at 7 to 63 mg/kg and 5 to 45 mg/kg dosage for patients weighing < 15 kg and ≥15 Kg, respectively [93]. Pregabalin may be considered as a second-line therapy when gabapentin is either poorly tolerated or ineffective [94]. For pregabalin, the usual initial pediatric dose for epilepsy ranges from 2.5 to 3.5 mg/kg per day in three divided doses, up to a maximum of 600 mg per day. The recommended dose is dependent upon body weight: 2.5 mg/kg/day for patients weighing 30 kg or more and 3.5 mg/kg/day for children with a weight below 30 kg.

Consensus guidelines widely recommend antidepressants as first-line therapy for either non-cancer-related [95] or malignant neuropathic pain in adults [96], while their efficacy and safety have not yet been validated in children [97]. However, similarly to adult patients, doses used against neuropathic pain are lower than those needed to achieve antidepressant effects; this helps maintain a safe profile, with possible side effects including anticholinergic manifestations, such as dry mouth, constipation, urinary retention, orthostatic hypotension, and even cardiac conduction abnormalities including prolonged QT interval, with risk of torsade de pointes and sudden cardiac death. Hence, before therapy starts, a baseline EKG is recommended [98]. Recommended duloxetine dosage in children 7 years of age and older is 30 mg once daily for 2 weeks, up to 60 mg once a day as a maximum dose.

Opioids are currently administered in the pediatric population for both acute and chronic pain management [99], starting from perioperative pain control, where they remain of great importance in the attempt to lower the risk for pain chronification, with a growing tendency to use combination and opioid-sparing therapies [100,101,102]. Although opioids are the main pharmacologic option in the case of chronic cancer pain in children and adolescents, high-quality evidence about chronic opioid therapies is lacking. Hence, they are widely administered only based on clinical knowledge and experience [103]. Adverse effects are still the leading cause for concern on their rational use; specific multidisciplinary settings were brought up for appropriate pain management, where opioid monitoring plays a pivotal role, particularly in patients with known respiratory comorbidities [94,104]. Most of the molecules on the market are not registered for use in the pediatric age. Therefore, in clinical practice, children are often treated with drugs that have only been studied and tested in adults, according to indications neither foreseen nor recorded for children (off-label use of drugs).

When opioids are deemed necessary, careful titration should be observed, starting from low doses and maintaining close monitoring for adverse effects [105]. As a first step, a weak opioid, such as tramadol, may be an option in case of moderate pain. Tramadol has a dual mechanism of action, acting both as an MOR agonist and inhibiting the reuptake of serotonin and noradrenaline [99,106], which may improve its efficacy in conditions of moderate-to-severe neuropathic pain. However, in 2017, the FDA restricted the use of tramadol for children below 12 years of age [107]. Soon after, in 2018, the EMA stated that tramadol is not recommended in children with breathing problems, such as obstructive sleep apnea (OSAS) and compromised respiratory function, since the symptoms of tramadol toxicity may be worse in these children. Despite restrictions, careful and rational use of tramadol is currently one of the most common pharmaceutical approaches to chronic pain in children. In children who are not fast metabolizers, the tramadol dosage of 1 to 5 mg/kg *ter in die* (T.I.D.) has been proven safe and effective for chronic pain management [93]. Other authors have suggested morphine as the best option, compared to tramadol, for moderate-to-severe chronic pain conditions in children [108]. A similar fate for the weak opioid codeine, which, after years of use among the pediatric population in combination with acetaminophen, as the only approved opioid, has also been restricted in children below 12 years in Europe for similar safety concerns, especially regarding respiratory depression [19]. Both these drugs, tramadol and codeine, indeed have a CYP450-mediated metabolism, which, due to genetic variability (poor vs. extensive metabolizers) and potential drug–drug interactions, may affect the efficacy and the tolerability profile. Recently introduced EMA regulatory measures have significantly decreased the prescription of codeine to patients below 12 years across Europe; however, the over-the-counter use of codeine, which may have occurred in France and the United Kingdom, could not be quantified [109]. In case of poorly controlled or severe pain, potent opioids may be necessary, including oxycodone, fentanyl, hydromorphone, and methadone. The aim is to establish an “around-the-clock” therapy to achieve the highest possible level of analgesia [110].

With special regard to cancer-related neuropathic pain in children, evidence on opioids’ efficacy and safety is particularly scarce [111]. Consequently, according to guidelines for adult patients [112], opioids are now recognized as second-line therapies for neuropathic pain by considering the deep concerns for their long-term neurocognitive effects, particularly on memory and learning [113]. Nonetheless, among potent opioids, low-dose methadone was found to be effective in neuropathic pain management in young patients with acute lymphoblastic leukemia, with an acceptable safety profile, with particular regard to cardiologic events [114]. Methadone has a unique pharmacodynamic profile that targets N-Methyl-D-Aspartate (NMDA) receptors, which may account for better efficacy in NP conditions [115]. However, methadone is not approved for children by either the EMA or the FDA.

Tapentadol is an atypical opioid with a dual mechanism of action, combining mu-opioid receptor (MOR) agonism and noradrenaline reuptake inhibition (NRI). Tapentadol was found to be effective and well tolerated in adults with neuropathic pain, both non-cancer- [116,117] and cancer-related [118]. The oral solution (OS) of tapentadol was authorized in the EU in 2018 as a treatment option for moderate-to-severe acute pain in hospital settings for children aged 2 to <18 [119]. Its efficacy in managing post-surgical pain was first assessed in patients aged 6 to <18 at 1 mg/kg dosage every 4 h [120]. Tapentadol was later found to be beneficial in controlling moderate-to-severe post-surgical pain in patients aged 2 to <18 years of age undergoing maxillofacial surgery, spinal, thoracic, and urologic surgery, and it was well tolerated at 1 mg/kg and at 1.25 mg/kg dosages, which were expected to produce exposure in a range similar to a 50 to 100 mg dosage of tapentadol IR in adults [121,122]. There is also recent evidence on the efficacy and safety of tapentadol OS e IV in children from birth to <2 years of age undergoing routine surgery at a weight-based dosage [123]. In addition, a prolonged release (PR) formulation of tapentadol is also available for long-term treatment in children with cancer and non-cancer pain. Howard et al. demonstrated its non-inferiority compared to morphine prolonged release (PR) for pain control at a starting dose of 1.25–1.5 mg/kg every 12 h, with a maximum dose of 4.5 mg/kg *bis in die* (B.I.D.), with optimal efficacy and safety profiles up to a 12-month period of therapy [124].

Buprenorphine is a semisynthetic opioid derived from thebaine, a partial MOR agonist (or a mixed agonist–antagonist) and KOR antagonist, with a limited maximum effect (ceiling effect) and the ability to bind to nociceptin receptor (NOP) [125]. Its role in managing CNCP [126] and cancer pain [127] was assessed. Regarding children and adolescents, buprenorphine may be administered via different routes: parentally, e.g., IV, for acute pain control, as in the emergency setting and for postoperative analgesia; the spinal and epidural route, for perioperative pain control; in contrast, the subcutaneous, oral, intranasal, and rectal routes are much less utilized. Most evidence is about the use of sublingual (SL) formulation and transdermal (TD) patches [128]. Transdermal formulation, in particular, was found to be effective in a single-arm non-randomized open-label trial for managing children’s cancer pain at 8.75, 17.5, and 35 mcg/hour dosages for patients weighing less than 15 Kg, between 15 and 30 kg, and more than 30 kg, respectively. TD buprenorphine was also well tolerated over extended treatment periods [129], even up to a 70 mcg/hour dosage. Overall, TD buprenorphine is still administered off-label in children with chronic non-surgical pain. Buprenorphine patches applied once every 72 h can be cut for better titration; weekly applied low-dose patches from 5 to 20 mcg/h are available [128]. TD buprenorphine has a good tolerability profile, albeit with possible local reactions, such as itching and erythema [130,131]. Sublingual buprenorphine is unavailable in all countries, and evidence is still scarce. However, it was found to be superior to morphine in terms of time to breakthrough analgesia for post-surgical pain, particularly after orthopedic and thoracic surgery, with a good safety profile for nausea, sedation, dizziness, pruritus, and respiratory depression [132]. Similarly, it was successful and safe in controlling pain in pediatric patients with chronic cancer [131] and SCD pain [133]. Given their unique pharmacodynamic properties, the aforementioned multi-mechanistic opioids, such as buprenorphine, tapentadol, and methadone, may represent a valid alternative to “traditional” potent opioids in case of opioid tolerance, as well as opioid-induced central sensitization and hyperalgesia in adults [134]; whether this is true for children and adolescents remains to be verified.

Among transdermal opioids, fentanyl has been safely used in a multimodal approach to pain management in children with cancer, at the mean dose of 33.2 mcg/h, in a range of 12 to 75 mcg/h with a 72 h rotation [92]. Transdermal fentanyl may be suitable in the pediatric population for the non-invasive route of administration [135]. Strong opioids, including transdermal formulations, are mainly used in the field of palliative care in children [136]. In the pediatric population from 2 to 16 years old, transdermal fentanyl can be used only in patients who are already receiving at least 30 mg of oral morphine equivalents per day (equivalent to a transdermal fentanyl dose of 12 mcg/h). From 45 to 134 mg/day of morphine, the conversion to TD fentanyl in children is 25 mcg/h. At doses greater than 25 mcg/h, the conversion from morphine is the same as it is for adult patients. TD fentanyl should not be used in children aged less than 2 years, because efficacy and safety have not been proven. No data are specifically available on the use of transdermal fentanyl for neuropathic pain in children with cancer. In general, transdermal opioids may be considered a good option for young patients with difficulty in swallowing.

Other adjuvant options for neuropathic pain management include NMDA channel blockers, such as ketamine; alpha-2-adrenergic agonists (e.g., clonidine or dexmedetomidine); and sodium-channel blockers, namely lidocaine. Ketamine is an anesthetic drug with analgesic properties. In adult patients, it is used for neuropathic pain management, given its antagonistic action on NMDA receptors [137] and probable effects on opioid receptors, as well as calcium or sodium channels [138]. Ketamine is administered at subanesthetic doses in pain therapy [139]. Although its use as sole therapy is possible, it is generally administered in combination with other analgesic drugs, namely opioids, especially in case of poor response, to reduce relative dosage and try to avoid opioid-induced hyperalgesia in adults [137]. A role for ketamine in neuropathic pain management is also possible in children with neoplasms [140,141]. Among alpha-2 agonists, dexmedetomidine has been used for pediatric analgosedation in different settings, from the emergency department [142] to intensive-care units [143] for years now. It is a highly selective α2 adrenergic receptor agonist with sedative and analgesic properties and minimal impact on the respiratory drive [144], all of which make it palatable for use in children, even at a very early age [145]. Its use as an adjuvant in the pediatric field for the management of neuropathic cancer pain was also assessed [146], always maintaining careful monitoring for possible side effects, namely hypotension and bradycardia [147].

Cannabis and medical marijuana are currently available in different forms; their role in neuropathic pain management in adults is currently under investigation as adjuvants in difficult-to-treat chronic pain syndromes [148]. The topic of cannabinoids for pain remains controversial. Nowadays, IASP guidelines give a weak recommendation against cannabinoid use for neuropathic pain in cancer patients [73], and ESMO guidelines have concluded that there is an unclear role of nabiximols as an add-on therapy in advanced cancer pain [149]. In the pediatric population, there is a lack of any valid evidence for chronic pain management against a proven potential correlation with mental illness, cognitive decline, and increased risk of addiction, which represent the main reasons for concern [150,151]. Consequentially, cannabis and marijuana should not be approved for use in people < 21 years of age, given that the structural and functional development of the human brain is ongoing up until the mid-20s [152]. Hence, the use of cannabis is not strictly recommended but should be carefully pondered on a case-by-case basis, especially in life-limiting conditions [153].

Palmitoylethanolamide (PEA) is a saturated fatty acid amide of palmitic acid, and it belongs to the ALIAmides family; despite having a chemical resemblance to anandamide, PEA has a lower affinity to cannabinoid receptors. It is implicated in neuroinflammatory conditions and nociception. It showed promising results in managing painful syndromes in adults [154] and children [155,156]. Possible preventing and analgesic effects of PEA in neuropathic cancer pain, especially CIPNs, have been assessed in both preclinical and clinical studies [157]. However, to the best of our knowledge, a possible role of PEA in cancer-related neuropathic pain management in children and adolescents is still a field for future investigation.

Lidocaine can be administered via the intravenous route for neuropathic cancer pain in children, mainly due to chemotherapy-induced peripheral neuropathy (CIPN), pain associated with vaso-occlusive crisis, and to manage refractory pain in advanced stages, with possible opioid-sparing purposes. However, IV administration requires unique settings and hemodynamic monitoring to intercept possible cardiovascular and neurological side effects [49,158]. Conversely, topical administration of lidocaine 5% patches could be considered a valid therapeutic alternative for localized neuropathic pain, with 12 h of applications per day [159]. The application of 8% capsaicin patches targeting TRPV1 activity has also been described in children with neuropathic pain [160].

Various non-analgesic drugs were evaluated to treat specific types of painful conditions in children, as is the case for bisphosphonates for bone pain [94]. Whether this applies to cancer pain remains to be evaluated. Neuromodulatory and interventional techniques may be an option in case of refractory cancer pain. For instance, deep brain stimulation targets areas associated with pain perception, namely centro-lateral (CL) and centro-medial parafascicular (CM-Pf) thalamic nuclei, the periaqueductal gray (PAG), the nucleus accumbens (NA), the posterior hypothalamus, the motor cortex, and so on [161]. Neurolytic blocks interrupt painful inputs from injured tissues to the spinal cord via applying chemical agents in the subarachnoid space [162]. Park et al. reported the use of scrambler therapy (ST) for a pediatric patient with leukemia suffering from neuropathic pain. With this technique, electrical non-painful stimuli are applied via electrodes attached along the dermatome of the painful area to obtain modulation of peripheral and central nervous structures [163]. Spinal cord stimulation (SCS) helps manage chronic pain by implanting percutaneous or paddle leads targeting the dorsal spinal columns to disrupt the pain signals traveling from the periphery to the brain [164]. Its validity in cancer neuropathic pain was assessed in adults [165], and its use in children and adolescents was also reported [166,167].

Transcutaneous electrical nerve stimulation (TENS) is based on applying electrical stimuli over the skin surface with various intensities, frequencies, and durations. Its efficacy in the management of non-cancer-related neuropathic pain in adults is widely demonstrated [168], and possibilities have surfaced that it may help control CIPNs in adults [169]; nonetheless, evidence on the use of TENS in pediatric pain is limited to non-malignant and periprocedural pain [170,171].

Besides their role in non-malignant pain management, peripheral nerve blocks (PNBs) may help reduce the risk of chronic post-surgical neuropathic pain in children and adolescents undergoing limb-sparing surgery or limb amputation for lower extremity malignancies [172].

## 5. Managing Anxiety and Emotions in Children and Adolescents with Cancer

In the last few years, along with the progress in therapy and the increased survival rate, the importance of a multidisciplinary and comprehensive approach to pediatric cancer pain treatment has emerged [173]. In the pediatric oncological population, a complex constellation of symptoms can be observed, including psychological manifestations such as anxiety, depression, low self-esteem, and social difficulties, alongside physical impairment, often presenting with pain, fatigue, and decreased autonomy [174,175,176]. In particular, an increased prevalence of depressive symptoms and anxiety has been demonstrated in children and adolescents with cancer [177,178] with persistence in their adult lives [179,180]. These psychological repercussions seem to correlate with poorer outcomes in terms of pain [179] and a poorer health-related quality of life (HRQOL) in pediatric cancer adult survivors concerning their healthy siblings [180]. A strict connection between depression, anxiety, and chronic pain has been observed in children [181], and it seems to be correlated to shared underlying neuronal circuits, specifically, the corticolimbic system involving the prefrontal cortex, the amygdala, and the hippocampus [182,183]. Furthermore, concerning neuropathic pain in the pediatric population, often associated with cancer, it has been shown that high levels of pain-related fear can negatively affect treatment outcomes. In contrast, a decrease in such symptomatology correlates with a reduction in disability and depression severity [181]. Hence, the importance of a combined integrative intervention has emerged in recent years, with recent evidence demonstrating its effectiveness not only in ameliorating the psychological impairment in this population but also in pain management, with a positive impact on their quality of life [173,184,185].

Among the primary non-pharmacological therapies explored in pediatric oncology are creative art, musical therapy, virtual reality, yoga, animal-assisted therapy (AAT), psychotherapy, and many others. For example, a combined mindfulness-based plus music therapy approach is practical in ameliorating pain levels, anxiety, and sleep disturbances in adolescents and young adults with osteosarcoma [186]. Beneficial effects have also been obtained with cognitive–behavioral therapy, especially concerning anxiety, depression, and pain [187], and it has been shown to promote a positive attitude and increase resilience with a consequent reduction in perceived distress and an overall improved well-being [188]. Moreover, in children undergoing hematopoietic stem cell transplantation (HSCT), problem-solving training (PST) has obtained favorable outcomes in terms of both psychological symptoms and pain reduction with adequate repercussions on these patients’ mental health [189].

Art therapy, including painting and handcrafting, especially in groups, has been a valid supportive treatment regarding reported physical energy, social relations, decreased stress levels, and negative feelings, with generally enhanced life and health status [190]. Regarding musical therapy, several studies have demonstrated its usefulness in reducing pain, fear, and anxiety, especially during procedures including chemotherapy, central radiotherapy [191], lumbar puncture [192], and HSCT [193]. Nevertheless, musical interventions have also been shown to have positive long-term therapeutic effects on such symptomatology, with beneficial repercussions on the patients’ overall health [191,194]. Similar results in reducing procedure-related pain, fear, and anxiety have been obtained via virtual reality during needle-inserting procedures [195,196] and during chemotherapy [197], with positive implications also on the caregivers’ reported procedural anxiety [198]. An improvement in the quality of life in the pediatric oncological population has additionally been observed, both on a physical and a psychological level, via the introduction of yoga into the rehabilitation program [199], with evidence of a reduction in pain scores in children and anxiety scores in adolescents [200]. Furthermore, beneficial effects on these patients’ emotions have been reported via the implementation of Make a Wish intervention in children with life-threatening cancer, with improvement in their quality of life in terms of emotional distress, depressive symptoms, and anxiety.

Finally, animal-assisted therapy (AAT) has been shown to minimize pediatric patient distress even in the once-hematological setting, particularly during painful procedures [201]. Numerous studies evaluated in a recent meta-analysis support using AAT to reduce pain and control blood pressure and heart rate in hospitalized children and teenagers [202]. Complementary therapies, such as music, play, pets, and art, utilized alongside conventional medical treatment should be encouraged to mitigate pain and anxiety in hospitalized pediatric patients [203].

Despite the heterogeneous data, there is ever-increasing and promising evidence of a multidisciplinary approach’s positive and beneficial effects on the oncological pediatric population. These comprehensive interventions should be aimed not only at treating the underlying pathology but at addressing every aspect of these patients’ lives to improve their general well-being in terms of quality of life and mental health both in the children undergoing treatment and in the survivors.

## 6. Conclusions

Neuropathic pain in children and adolescents with cancer is the result of multiple causes; hence, its evaluation is laborious. Young patients may find it challenging to describe their pain thoroughly in qualitative and quantitative aspects. Evidence from the literature on this matter is scarce, primarily because of the difficulty of setting up RCTs for young patients. Moreover, such scores cannot replace morphometric, neurophysiological, and psychophysical techniques as gold standards for a definite diagnosis of neuropathic pain. Nonetheless, such tests are not always promptly available.

Neuropathic pain management is still challenging in children and adolescents for different reasons. Firstly, most of the available drugs for pain management in adults are not approved in childhood, and pediatricians should be confident enough with pain treatments to use these molecules off-label in the pediatric setting. Secondly, most of the available analgesics may be associated with adverse effects, often leading to therapy discontinuation and scarce pain control. Thirdly, specific recommendations or guidelines still need to be made available. Hence, drug prescriptions and administration are based on clinical practice and data derived from studies on adults. Finally, treating a child in pain often means treating not only a single patient but the entire family. Parents mediate pain perception and acceptance of the proposed treatments in young children. For all these difficulties and many others, a multidisciplinary approach, with early identification of patients with chronic pain, is crucial. Specialists trained in pediatric pain treatment are essential for appropriately managing this vulnerable population [204].

Further research should focus on validating specific assessment tools for the evaluation of neuropathic pain in children and adolescents, on promoting RCTs in children to provide better quality data, as well as finding new therapeutic targets to reduce drug-induced adverse effects, which may worsen the quality of life and the overall health status of young patients facing against cancer.

## Figures and Tables

**Table 1:** Common pediatric oral analgesics.

	P.O. Dose/Starting Dose	EMA (Europe) Approved	FDA (US) Approved
**NSAIDs/APAP**			
Ibuprofen	5–10 mg/kg q 6–8 h	3 mo	6 mo
Naproxen	5 mg/kg q 8–12 h	1 year and > 10 kg	2 years (rheumatoid arthritis)
Acetaminophen	10–15 mg/kg q 6 h	Neonates	Neonates
**Corticosteroids**			
Dexamethasone	0.25 mg/kg q 6 h	Neonates	Neonates
**Opioids**			
Codeine/acetaminophen	30/500 mg q 6 h	12 years	12 years
Tramadol	1–2 mg/kg q 6 h (1–12 years) 50 mg q 4–6 h (≥12 years)	1 year and >10 kg	12 years
Morphine	0.3 mg/kg q 3–4-h	Neonates	Not authorized
Oxycodone	0.1–0.15 mg/kg q 4–6 h	12 years	11 years (extended release)
Tapentadol	1.25 mg/kg q 4 h	2 years	Not authorized
TTS Fentanyl	12 mcg/h every q 72 h	2 years	2 years
TTS Buprenorphine	5 mcg/h q 7 d	18 years	16 years (subdermal implant for opioid dependence)
**Anticonvulsants**			
Gabapentin	5–40 mg/kg/day in 3 divided doses	6 years	3 years (partial seizures)
Pregabalin	2.5–3.5 mg/kg/day in 3 divided doses	Not authorized	1 mo (partial seizures)
**Antidepressants**			
Duloxetine	30 mg/day, 60 mg/day after 2 weeks	Not authorized	7 years (generalized anxiety disorder), 13 years (fibromyalgia)
